# Coco Peat as Agricultural Waste Sorbent for Sustainable Diesel-Filter System

**DOI:** 10.3390/plants10112468

**Published:** 2021-11-16

**Authors:** Gayathiri Verasoundarapandian, Nur Nadhirah Zakaria, Noor Azmi Shaharuddin, Khalilah Abdul Khalil, Nurul Aini Puasa, Alyza Azzura Azmi, Claudio Gomez-Fuentes, Azham Zulkharnain, Chiew Yen Wong, Muhammad Fahdli Rahman, Siti Aqlima Ahmad

**Affiliations:** 1 Department of Biochemistry, Faculty of Biotechnology and Biomolecular Sciences, Universiti Putra Malaysia, Serdang 43400, Selangor, Malaysia; gayathiri1802@gmail.com (G.V.); nadhirahairakaz@gmail.com (N.N.Z.); noorazmi@upm.edu.my (N.A.S.); nurulainipuasa@gmail.com (N.A.P.); 2 Institute of Plantation Studies, Universiti Putra Malaysia, Serdang 43400, Selangor, Malaysia; 3 School of Biology, Faculty of Applied Sciences, Universiti Teknologi MARA, Shah Alam 40450, Selangor, Malaysia; khali552@uitm.edu.my; 4 Faculty of Science and Marine Environment, Universiti Malaysia Terengganu, Kuala Nerus 21030, Terengganu, Malaysia; alyza.azzura@umt.edu.my; 5 Department of Chemical Engineering, Universidad de Magallanes, Avda. Bulnes, Punta Arenas 01855, Chile; claudio.gomez@umag.cl; 6 Center for Research and Antarctic Environmental Monitoring (CIMAA), Universidad de Magallanes, Avda. Bulnes, Punta Arenas 01855, Chile; 7 Department of Bioscience and Engineering, College of Systems Engineering and Science, Shibaura Institute of Technology, 307 Fukasaku, Minumaku, Saitama 337-8570, Japan; azham@shibaura-it.ac.jp; 8 School of Health Sciences, International Medical University, Bukit Jalil, Kuala Lumpur 57000, Malaysia; wongchiewyen@imu.edu.my; 9 Merbau Agrotechnology (M) Sdn. Bhd., JA2391, Jalan Sungai Renggi 2, Kampung Sungai Renggi, Merlimau 77300, Melaka, Malaysia; merbau.agrotechnology@gmail.com; 10 Laboratory of Bioresource Management, Institute of Tropical Forestry and Forest Products (INTROP), Universiti Putra Malaysia, Serdang 43400, Selangor, Malaysia

**Keywords:** diesel spills, agriculture waste, coco peat, biosorbent, filter system

## Abstract

Oil spill incidents are hazardous and have prolonged damage to the marine environment. Management and spill clean-up procedures are practical and rapid, with several shortcomings. Coco peat (CP) and coco fibre (CF) are refined from coconut waste, and their abundance makes them desirable for diesel spillage treatment. Using a filter-based system, the selectivity of coco peat sorbent was tested using CP, CF and peat-fibre mix (CPM). CP exhibited maximal diesel sorption capacity with minimal seawater uptake, thus being selected for further optimisation analysis. The heat treatment considerably improved the sorption capacity and efficiency of diesel absorbed by CP, as supported by FTIR and VPSEM–EDX analysis. Conventional one-factor-at-a-time (OFAT) examined the performance of diesel sorption by CP under varying parameters, namely temperature, time of heating, packing density and diesel concentration. The significant factors were statistically evaluated using response surface methodology (RSM) via Plackett–Burman design (PB) and central composite design (CCD). Three significant (*p* < 0.05) factors (time, packing density and diesel concentration) were identified by PB and further analysed for interactions among the parameters. CCD predicted efficiency of diesel absorbed at 59.92% (71.90 mL) (initial diesel concentration of 30% *v*/*v*) and the experimental model validated the design with 59.17% (71.00 mL) diesel sorbed at the optimised conditions of 14.1 min of heating (200 °C) with packing density of 0.08 g/cm^3^ and 30% (*v*/*v*) of diesel concentration. The performance of CP in RSM (59.17%) was better than that in OFAT (58.33%). The discoveries imply that natural sorbent materials such as CP in oil spill clean-up operations can be advantageous and environmentally feasible. This study also demonstrated the diesel-filter system as a pilot study for the prospective up-scale application of oil spills.

## 1. Introduction

Natural organic sorbents from agriculture biomass or by-products have received attention as they create a substantial possibility for highly effective oil separation from water [1,2,3]. Some of the lignocellulosic sorbent materials are peat moss, coconut pith, cotton, palm oil empty fruit bunch or leaves, kapok, sugar cane bagasse, corn cob, sponge gourd fibre, rice straw and fruit peels [4,5,6,7,8,9]. Apparent disadvantages such as non-biodegradability, recyclability and low buoyancy constrain their use. 

Physical, mechanical and biological clean-up techniques are typically adopted as the recovery measure for oil spills but are potentially destructive to the aquatic environment. The implementation of agricultural wastes and their residual products as potential spill clean-up alternatives is known to be inexpensive, freely available and almost 100% biodegradable [10]. Current practices of spill clean-up involve absorbent booms with a combination of synthetic and some natural sorbent materials [11]. Agriculture waste is often susceptible to bacteria or fungi infections; thus, necessary pre-treatments are required, such as physical (heat) and chemical (solution-based) treatments, preventing secondary contaminations [12,13,14]. The application of sorbent materials for oil removal is appealing partly because of their capacity to convert oil contaminants in the liquid state into solid or semi-solid states [15,16]. 

Coconut (*Cocos nucifera*. L) is a tropical fruit belonging to the family *Arecaceae* (palm family) found in abundance in several Asian nations especially India, the Philippines, China, Thailand and Malaysia. By 2019, its production reached 0.54 million metric tonnes and 30 thousand metric tonnes of consumption in Malaysia. After oil palm, rubber and rice, coconuts are Malaysia’s 4th and the world’s 12th most important industrial commodity. The majority is locally concentrated in Sabah and Sarawak and is also an essential food industry. A huge amount of coconut biomass could be generated from coconut husk and coconut juice, producing up to 50,000 tonnes and 182,000 m^3^ based on a study in Thailand [17]. Coconut fruits have significant economic value, dominating the food, pharmaceutical, textile and agricultural industries. Excessive consumption of coconut creates a substantial percentage of coconut residue, increasing the pressure on Malaysia’s landfills, as reported by the Malaysian Agricultural Research and Development Institute (MARDI). Typically, a mature coconut palm could produce 47–50% of husks and 14–15% shell wastes. Obeng et al. [14] also indicated that husk and shell wastes account for 62–66% of a whole coconut, which might be regarded as a useful resource. 

Coco peat (CP) or coir pith and coconut fibre (CF) is a natural by-product from coconut husks processed through post-harvesting. CP is commonly available for agriculture and horticulture applications, whereas CFs are used in construction or textile industries [18]. CP offers many underutilised benefits such as its low cost, degradability, availability and recyclability; hence, it is critical to investigate its ideal use for environmental sustainability. The following are the composition of raw CP: 35.0% cellulose, 25.2% lignin, 7.5% pentosans, 1.8% fats and resins, 8.7% ash content, 11.9% moisture content and 10.6% other substances [19,20,21,22]. CP makes a great sorbent due to its constituents such as hydroxyl, carboxyl, ether, phosphate and the presence of amino groups [23]. 

An excellent oil sorbent must have a great affinity for absorption or adsorption, polar and oleophilic characteristics, as well as uniform dispersion in enclosing liquid oil in the interior of its distinctive structure. The outstanding aptitude of lignocellulose-based materials of CP to absorb oil rapidly as well as the intense capillary tension between oil and lignocellulose have been ascribed to much research [24,25]. Lignocellulose holding a variety of reactive functional groups allows for extensive and rapid absorption of oil and water [26]. Additionally, coir fibre has been previously shown to perform crude oil absorption successfully [27,28]. The high content of lignin in CP allows it to be resistant to biological degradation and biodegradable, and it requires up to two decades to decompose completely due to its durability. Pyrolysed activated carbon and chemically modified CP/CF have been demonstrated in the past in effective oil clean-up [19,20,27,29,30,31]. However, such treatments are not eco-friendly, are hazardous and have a high cost. Surface modifications via physical treatment such as thermal heating are convenient, cost effective and harmless to the environment. Treated waste sorbents would not produce organic compounds or create high biological oxygen demand, chemical oxygen demand and total organic carbon in contaminated waters [32,33]. Furthermore, heat-treated sorbent materials have great mechanical and thermal stability. Due to its porous and hydrophobic properties, the sorbent is readily reused by burning, distillation or compression [34,35].

The physical properties of CP are highly dependent on the processing technique. It has a very high water-holding capacity, and its use as a sorbent for pollutant (heavy metals, dyes, phenolic compounds and radionuclides) removal has also been studied [17,28,36,37,38,39]. CP is available in dust particles, which requires extra care when handling, particularly considering the enormous surface area of water. Therefore, several systems are applied to remove oil from water, such as separators or filters, aerogels and gelators. Yang et al. [40] proposed using the magnetism hypothesis for better recovery of absorbed by magnetised CP. According to investigations, a straightforward pressing technique is adequate to absorb the remainder of the oil sorbed by the fibres, providing the sorbents to be reused for oil spill clean-up multiple times [29]. Moreover, filtered-absorbed oil sorbents would be disposed of following the United States Environmental Protection Agency (EPA) guidelines and allowable fuels to be recycle [41]. After several uses, the sorbent could be used as a landfill or disposed by incineration. Over time, the sorbent will disintegrate entirely without creating harmful by-products [42].

Oil spill management of marine waters remains a major concern. In the present context, the capability of CP as an oil-sorbent material is untapped as a prospective application of spill clean-up via a filter-based oil sorption system. This study investigates the usage of CP as an agricultural waste for diesel spill clean-up from the seawater ecosystem by utilising a filter-based system. In addition, the statistical optimisation of numerous aspects affecting the efficiency of diesel and seawater absorption was performed via One-Factor-At-a-Time (OFAT) and Response Surface Methodology (RSM).

## 2. Results and Discussion

### 2.1. Screening of Different Types of CP Sorbent Materials

The sorption capacity (SC) and efficiency of diesel absorbed were determined for untreated (UNT) and treated (T) samples of CP, CF and CPM. Assessing real-life circumstances of oil contamination often involves both oil and seawater. The diesel and seawater values were considered throughout this study to improve the validity of potential pilot tests. For CP samples, the most excellent sorbent was chosen based on the high efficiency of diesel and the low amount of seawater absorbed.

**Figure 1:**
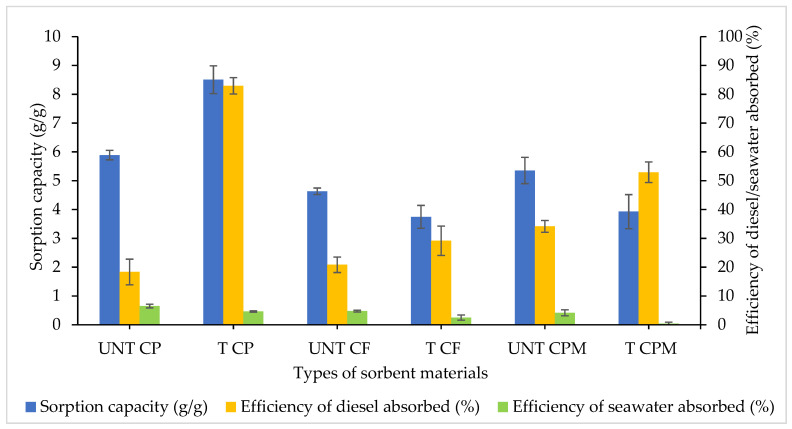
Screening for CP sorbent materials. UNT: untreated, T: treated, CP: coco peat, CF: coco fibre and CPM: coco peat mix (1 peat: 1 fibre). The error bars represent the mean ± SEM for three replicates (*n* = 3).

From Figure 1, treated CP absorbed diesel most efficiently with the highest sorption at 82.92% (*p* < 0.0001) and an SC of 8.50 g/g in the presence of 10% (*v*/*v*) diesel and seawater mixture. Likewise, the effectiveness in diesel sorption demonstrated by UNT CP was 18.33% and the SC was at 5.89 g/g. There were significant differences confirmed between UNT CP and T CP samples in terms of their SCs (*p* = 0.0013) and efficiency of diesel absorbed (*p* <0.0001) but no notable differences for absorbed seawater (*p* = 0.2141). 

For UNT CF and T CF sorbents, there were no significant differences identified for SC (*p* = 0.4501), efficiency of diesel (*p* = 0.3955) and seawater (*p* = 0.1000) absorbed. Even though the SC for UNT CF (4.63 g/g) was higher than that for T CF (3.75 g/g), the efficiency of diesel absorbed for UNT CF (20.83%) was lower than that of T CF (29.17%). 

T CPM exhibited moderate performance of diesel absorbed with 52.92% efficiency but showed lower efficiency for UNT CPM at 34.17% (*p* = 0.0073). Both SCs showed no significant differences (*p* = 0.0835) for T CPM (3.75 g/g) and UNT CPM (3.93 g/g). It was identified that the T CPM demonstrated the least efficiency in seawater absorbed at 0.42% compared with UNT CPM at 4.17% (*p* = 0.0038). An ANOVA analysis indicated the significant differences observed for the SCs (F_5,12_ = 28.01, *p* < 0.0001) and the efficiency of absorbed diesel (F_5,12_ = 69.04, *p* < 0.0001) and seawater (F_5,12_ = 15.11, *p* < 0.0001) between the CP sorbent materials.

It could be challenging for CP to acquire excellent characteristics owing to its high moisture sorption, approx. 200% of its weight. Treating them with heat or chemical substances greatly improves their sorption capacity and minimises water uptake [28]. Coconut husk (fibre) has previously exhibited a sustainable biosorbent for oil removal through various modifications. Even though it has a strong oil sorption capacity, it has a relatively high efficiency of water absorbed, which might be a major constraint in practical implementation [19,27,29,30,43]. CP pith/peat has therefore been overlooked for its oil sorption capabilities. Still, it is best recognised for its use as a modified biosorbent for dyes, metal ions, phenolic compounds and wastewater treatment [44,45,46,47]. Following that, T CP was preferred to optimise various factors affecting diesel sorption due to their excellent diesel and poor seawater sorption efficacy.

### 2.2. Characterisation of Biosorbent

#### 2.2.1. FTIR Spectrum Analysis of CP

**Figure 2:**
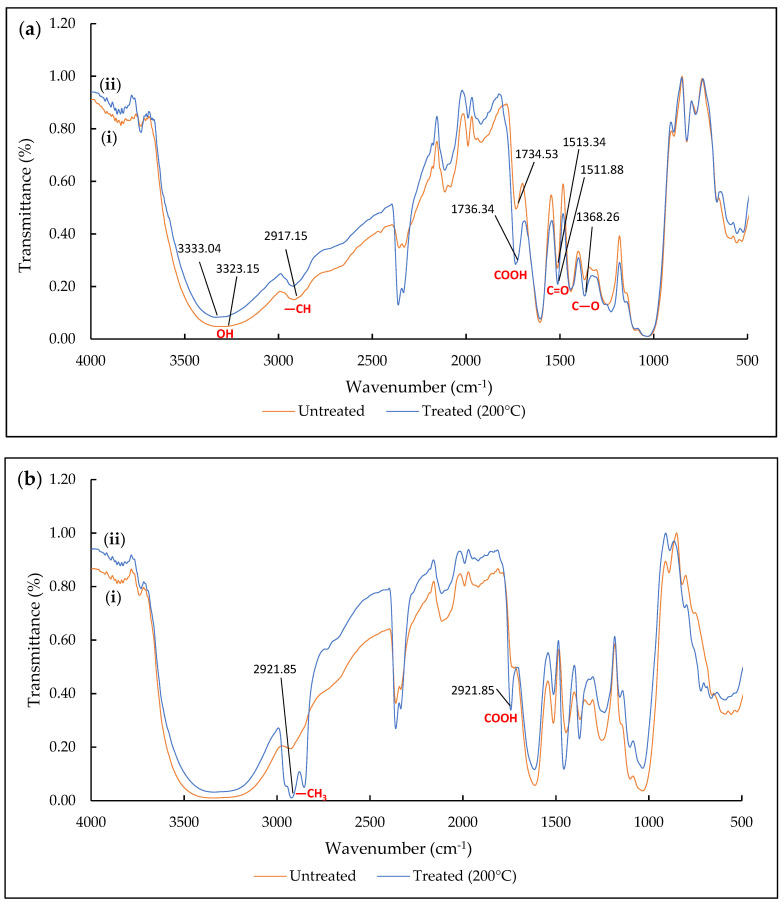
FTIR spectrum of CP samples (**a**) before filtration and (**b**) after filtration: (**i**) untreated and (**ii**) treated at 200 °C.

The FTIR spectra of UNT and T CP (200 °C) samples were examined and compared before and after the filtration process (Figure 2). The correlation between the functional groups and the effect of heat treatment as well as the diesel–seawater sorption could be deduced from the analysis. The characteristics of the CP are inclusive of cellulose, hemicelluloses and lignin, which are attributed to functional groups (C–O, C=O, C–H and O–H) [41].

Figure 2a presents the spectrum for UNT CP and T CP before filtration by the diesel–seawater mixture. Broad peak stretching of the hydroxyl group was observed at 3333.04 cm^−1^ (T CP) and 3323.15 cm^−1^ (UNT CP). The hydroxyl group present in cellulose was reflected in the peak. The stretching vibration of the CH alkyl group (fingerprint region) was only detected at 2917.15 cm^−1^ for UNT CP. The aliphatic nature of CP suggested that the stretching mode of CH contributes to the hydrophobicity and oleophilic properties [47,48]. The carboxyl group indicated a peak at 1734.53 cm^−1^ in untreated CP, which then increased in T CP (1736.34 cm^−1^). The bending of cellulose and hemicellulose (stretching C–O) in T CP was distinct 1368.26 cm^−1^ but absent in UNT CP. The stretching of C=C in the aromatic compounds reflects the lignin, where the peak intensity for T CP (1511.88 cm^−1^) was slightly lower than that for UNT CP (1513.34 cm^−1^), indicating the loss of lignin through heat treatment on CP. The deformation of C–O was detected between the region 1227.28–823.82 cm^−1^ for T CP, which is lower compared with the UNT CP peak at 1245.67–824.07 cm^−1^.

Figure 2b reveals the FTIR spectrum for CP samples after diesel–seawater treatment. The stretching of the alkyl group was wider after filtration. This peak is relatively associated with the alkyl (CH_3_) long chain of diesel hydrocarbons, which is greatly reflected in T CP (2921.85 cm^−1^) [49]. The ester linkage of the carboxyl group was identified at 1742.83 cm^−1^ for T CP, but none was detected for UNT CP.

#### 2.2.2. Surface Morphology Analysis

**Figure 3:**
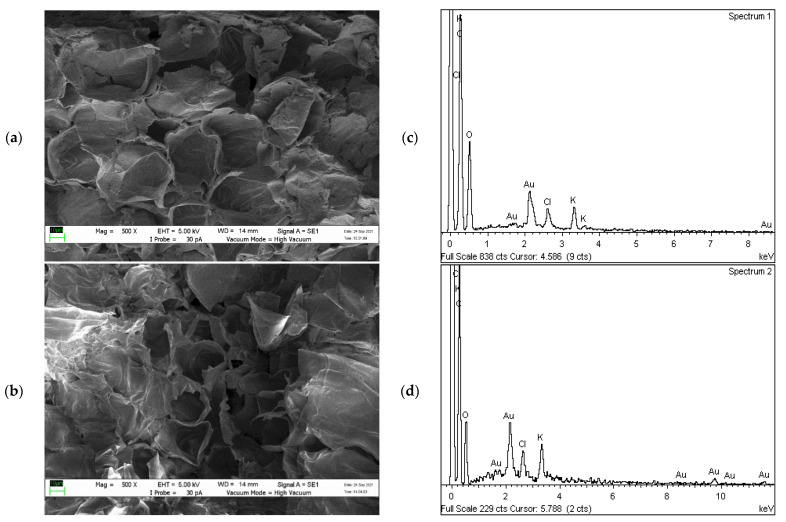
SEM and EDX analysis before filtration: (**a**,**c**) UNT CP and (**b**,**d**) T CP.

**Figure 4:**
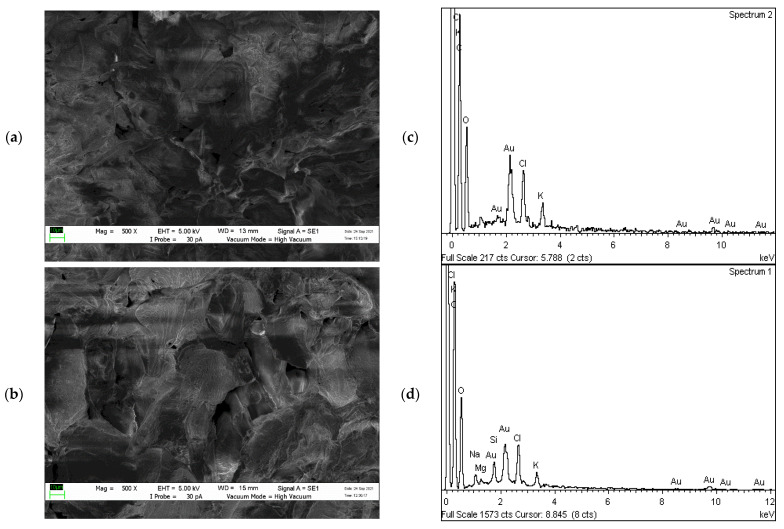
SEM and EDX analysis after filtration: (**a**,**c**) UNT CP and (**b**,**d**) T CP.

The surface morphological characterisation of UNT and T CP samples is presented in Figure 3 and Figure 4, before and after filtration of diesel–seawater, observed using SEM. Figure 3a shows the CP before heat treatment and has complex, irregular and large pores. After heating at 200 °C, the surface of CP was significantly ruptured and the pores appeared more hollow and darker (Figure 3b). A large amount of potassium was detected in T CP as CP is generally abundant in potassium ions (2.4 wt. % and 0.02 at. %) (Figure 3d) [50]. The thermal treatment substantially stripped the waxy thin layer formed by lignin, cellulose and hemicellulose on the CP surface [51,52]. The perforated porous region of T CP may have contributed to the enhanced efficiency of diesel–seawater molecule sorption. Figure 4b shows the slick oily layer covering the internal porous region of T CP due to the large and exposed surface area. The EDX revealed that sodium (2.26 wt. % and 0.03 at. %), magnesium (0.29 wt. % and 0.003 at. %), silica (3.91 wt. % and 0.04 at. %) and chlorine (10.46 wt. % and 0.08 at. %) were present after the treatment. The low efficiency of diesel–seawater sorption by UNT CP was contributed by their enclosed pores and active sites (Figure 4a). The presence of chloride ions indicated high seawater sorption by UNT CP. A previous absorption study reported that efficient absorption of diesel molecules into the surface and porous structures of CP was determined by the relative porous surface that had apparent macropores [27]. However, in this study, only qualitative observations were made using SEM and detailed data such as porosity measurement, pore volume distribution, diameters and surface area of CP are needed to verify the contribution of surface characteristics towards improving sorption efficiency.

### 2.3. Optimisation of Diesel–Seawater Sorption Conditions via OFAT

#### 2.3.1. Effects of Heat Treatment

**Figure 5:**
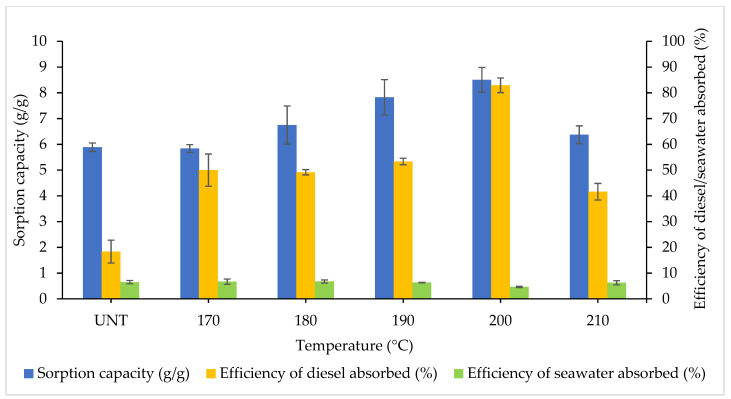
Effects of temperature on sorption capacity and efficiency of diesel–seawater absorbed by CP. The error bars represent the mean ± SEM for three replicates (*n* = 3).

Figure 5 presents the SC and efficiency of diesel–seawater absorbed at varying heating temperatures. The efficiency of diesel absorbed increases as the temperature rises but declines as it reaches maximal temperature. At the heating temperature of 200 °C, the highest SC was observed at 8.50 g/g and diesel was absorbed with an efficiency of 82.92%. CP heated at 200 °C was significantly different when compared with UNT CP (*p* < 0.0001) for diesel sorption. However, no significant difference was identified for seawater sorption (*p* = 0.1954), even though CP treated at 200 °C absorbed the least amount of seawater. The UNT CP (18.33%) remained the least effective as a biosorbent (*p* = 0.0160), and at 210°C, the efficiency of diesel absorbed fell by two-fold at 210 °C (41.6%). The overall SCs (F_5,12_ = 3.423, *p* = 0.0375) and efficiency of diesel absorbed (F_5,12_ = 26.03, *p* < 0.0001) by CP had a significant difference between ranging temperatures. ANOVA also identified no significant difference in the efficiency of seawater absorbed between the treatments (F_5,12_ = 2.231, *p* = 0.1183). 

The enhancement of physical contact between binding sites of diesel fuel molecules and CP was attributed to the decline in hydrogen bonds and Van der Waals interactions at higher temperatures. Conversely, a drop in temperature is attributable to a reduction in the overall biosorbent contact area and a decline in the accessibility to the sorption sites. The diffusion rate of diesel oil droplets through the outer fluid layer and the inner pores of the CP particle was also enhance as the temperature increased. Thus, the solubility of diesel fuel in seawater improves with a reduction in the mobility of diesel fuel species in the solution [30,50,51]. Chemical modifications and pyrolysis treatment are considered costly and complex and may introduce harmful substances to the water system [52].

#### 2.3.2. Effects of Heating Time

**Figure 6:**
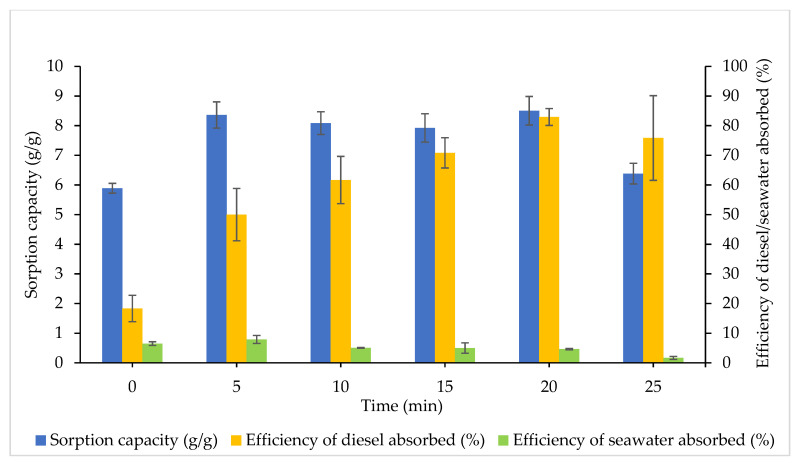
Effects of heating time. The error bars represent the mean ± SEM for three replicates (*n* = 3).

According to Figure 6, the optimal temperature at 200 °C was selected to study the effects of different heating times. The efficiency of diesel absorbed by CP improved when the heating duration was increased. However, there was a drastic reduction at 210 °C. Besides 5 min, which demonstrated maximum seawater sorption, the efficacy of seawater absorbed declines as the heating time increases. The most preferable heating time was observed at 20 min with the highest SC (8.50 g/g) and efficiency of diesel absorbed (82.92%), with the lower seawater absorbed (4.62%). When compared with 0 min, there was a significant difference for SC (*p* = 0.0011) and diesel sorption efficacy (*p* = 0.0002). Overall ANOVA found significant differences between the heating time for SC (F_5,12_ = 11.55, *p* = 0.0003), efficiency of diesel (F_5,12_ = 12.30, *p* = 0.0002) and seawater (F_5,12_ = 7.246, *p* = 0.0052) sorption.

The thermal modifications are necessary to ensure that the moisture content of CP is reduced to the desired level. It is critical to enhance the diesel sorption, which is essential in oil spill clean-up [53]. The basic method of drying coir pith/peat is to expose it to the sunlight and to allow spontaneous evaporation to ensue. It is indeed a cost-effective heating technique but has several shortcomings, including being laborious and time-consuming, uncontrollable weather conditions, interference by insects and the need for vast working space [54,55]. Meanwhile, industrial or laboratory approaches rely on hot air and oven drying for several conditions, notably rapid and even heating. Fernado et al. [50] proposed that the moisture content of coco pith/peat was significantly reduced as the duration of heating increased using a laboratory hot air dryer. The optimal temperature was observed at 140 °C for 1 h 45 min, and a higher temperature correlates with shorter heating time (200 °C at 47 min). Heating with a longer duration generally improves the hydrophobicity of CP, which enhances the binding of oil molecules and sorption capacity. However, the present study suggests that heating at 200 °C for 20 min significantly enhances the effectiveness of diesel absorbed, as a longer heating time (>20 min) greatly reduces the lignocellulosic materials [50,56].

#### 2.3.3. Effects of Packing Density in the Filter Spacer Column

**Figure 7:**
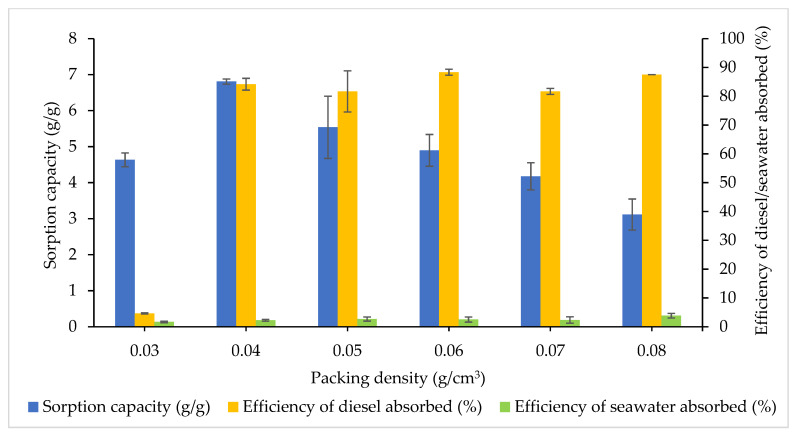
Effects of the packing density of CP. The error bars represent the mean ± SEM for three replicates (*n* = 3).

The packing densities (0.03–0.08 g/cm^3^) affecting the sorption capacity and efficiency of diesel–seawater sorption were investigated using a spacer mesh net attached to the filter column (Figure 7). There was a significant difference in SC between the treatments (F_5,12_ = 3.491, *p* = 0.0353) and the efficiency of diesel (F_5,12_ = 3.491, *p* = 0.0353), but no significance was indicated for the seawater absorbed (F_5,12_ = 4.298, *p* = 0.2973). As the packing densities increased, the SC of CP increased from 0.03 g/cm^3^ to 0.04 g/cm^3^ (*p* = 0.5164), followed by a declining trend, but fluctuation was observed for the efficiency of diesel absorbed. A packing density of 0.06 g/cm^3^ (88.33%) demonstrated the most efficacy in diesel sorption for CP even though no significant difference was shown for 0.04 g/cm^3^ at 84.17% (*p* = 0.8610) and 0.08 g/cm^3^ with 87.50% sorption (*p* = 0.9999). To determine the optimal packing density as a working sample, the efficiency of seawater absorbed should be at a minimum. As a result, the seawater sorbed for a packing density of 0.08 g/cm^3^ (3.83%) had the maximum efficiency compared with 0.06 g/cm^3^ (2.50%) and was ruled out as a suitable packing medium for CP (*p* = 0.6454).

Notably, the availability of more active sites on the CP surface indicates long chain groups of acyl present, which promote the efficiency of diesel absorbed with increasing packing density. A grounded biosorbent can exhibit greater oil sorption capacity due to the exposed contact surface and binding regions, allowing smaller sorbent particles to access [25]. Hence, the interaction between the polar oil molecules and the sorbent effectively increases [28,47]. When sorbent materials are manually compressed, their packing density qualities are strengthened with better oil sorption capacity. Thus, high packing density indicates a lower capacity of oil sorption and vice versa [27,57]. This is in agreement with the efficiency of diesel absorbed at a packing density of 0.06 g/cm^3^.

#### 2.3.4. Effects of Diesel Concentration

**Figure 8:**
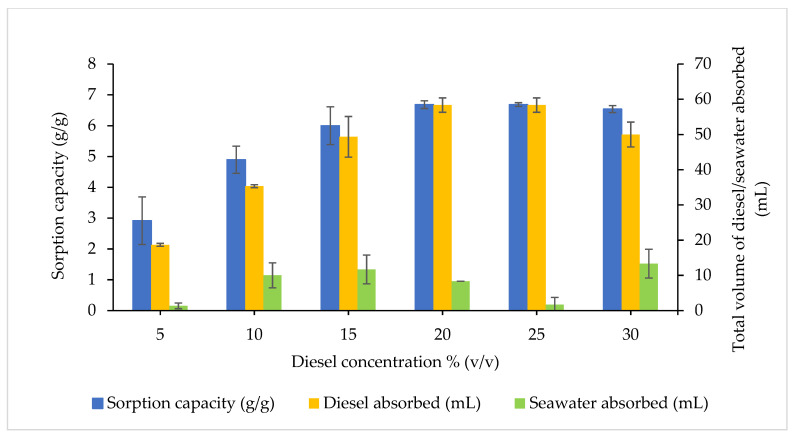
Effects of diesel concentrations based on their sorption capacity (g/g) and the diesel–seawater absorbed (g/mL). The error bars represent the mean ± SEM for three replicates (*n* = 3). Packing density constant at 0.06 g/cm^3^.

The effects of different initial diesel concentrations ranging from 5 to 30% (*v*/*v*) were investigated on the diesel–seawater sorption efficacy and SC, as depicted in Figure 8. The SC increased as the initial diesel concentration increased while the efficiency of the diesel absorbed (%) was reduced. An ANOVA analysis identified significant differences for the SC (F_5,12_ = 16.88, *p* < 0.0001) and efficiency of diesel absorbed (%) (F_5,12_ = 29.77, *p* < 0.0001) between the treatments influenced by the initial diesel concentration. However, no significant difference was evident for the efficiency of seawater absorbed (F_5,12_ = 1.512, *p* = 0.2575).

A low diesel concentration of 5% (*v*/*v*) infused with seawater demonstrated the highest efficiency of diesel absorbed at 93.33% (18.67 mL) with the lowest SC at 2.92 g/g when reached an equilibrium. Nonetheless, at 20% and 25% (*v*/*v*), the initial diesel concentrations had the highest SC at 6.68 g/g (*p* > 0.9999) whereas the efficiencies of diesel absorbed were 72.92% (58.33 mL) and 58.33% (58.33 mL), respectively (*p* = 0.1128). Apart from that, the seawater absorbed at 20% (*v*/*v*) was 8.33 mL (2.08%) and 25% (*v*/*v*) at 1.7 mL (0.41%) with no significant difference (*p* = 0.8571). The ability of CP to absorb diesel at the concentration of 15% (*v*/*v*) was 49.33 mL (82.22%), with the decreased SC at 6.00 g/g, and high seawater sorption was observed at 11.67 mL (2.92%).

These values were affected by the large surface-active areas exposed to diesel fuel, which led to more diesel oil molecules that bind and occupy the active sites on the CP surface at a low initial diesel concentration. Contrariwise, as the initial diesel concentration increases, the SC is reduced with a low efficiency of diesel absorbed. Low SC indicates that the active sites present for sorption were insufficient to contain the diesel fuel molecules. The saturation was achieved rapidly and most of the free oil molecules remained when the molecules were attached to the CP surface at a high diesel concentration [36,57,58]. Past investigations had reported similar evaluations on the effect of initial diesel concentration for coconut-based biosorbents [27,28,30]. Hence, considering both responses, the initial diesel concentration at 25% (*v*/*v*) was selected as the optimal initial diesel concentration to evaluate the significant factors and their interaction. 

### 2.4. Statistical Approach via Response Surface Methodology

#### 2.4.1. Plackett Burman Design

Table 1 displays the minimum and maximum experimental values obtained in 18 runs for diesel and seawater sorption efficiency. At run no. 6, the lowest experimental values acquired for diesel–seawater sorption were 53.67 mL (59.63%) and 8.33 mL (9.25%). The highest values obtained were at run no. 17 for diesel–seawater sorption, which were 70.33 mL (58.60%) and 5.00 mL (4.17%). 

The significant model terms were B (*p* = 0.0169), C (*p* = 0.0017) and D (*p* = 0.022) (Table 2), indicating that the factors including time, packing density and diesel concentration significantly affected the efficiency of diesel absorbed. The effect of temperature was excluded (*p* > 0.100) for corresponding CCD analysis. The validation of the model (*p* = 0.0018) was revealed through the R^2^ coefficient of determination at 0.7376, indicating that, with less fit and weak correlation of the experimental and predicted values, the regression analyses generated a quadratic equation for the responses:

Y = +35.56 − 5.77A − 9.33B + 13.56C + 8.83D(1)

#### 2.4.2. Central Composite Design (CCD)

The significant parameters chosen from PBD were further analysed using CCD to identify the optimal and interacting conditions between the factors. A total of 20 experimental runs were generated from three significant factors and followed by optimisation at the five-level CCD approach. Table 3 displays the experimental variables with the adjacent experimental and predicted values of diesel and seawater sorption by CP. 

The highest experimental and predicted values for diesel sorption were 76.00 mL with 84.44% efficacy and 76.81 mL (85.34%). The seawater sorption acquired for experimental value was 14.67 mL (3.67%), and the corresponding predicted values for seawater absorbed were at 13.79 mL (15.32%). The diesel sorption was observed at run No. 12, with a lower experimental value of 11.00 mL (12.22%) than the predicted values at 22.40 mL (24.89%). The experimental value (30.00 mL) obtained for seawater sorption was higher compared with the predicted value (24.01 mL). Table 4 with the ANOVA analysis confirmed that the model was highly significant (*p* = 0.0001). The factors packing density (B) and diesel concentration (C) were significant (*p* < 0.05) to the response. In this study, the most significant factor is packing density, in which CP was packed in the spacer column within the filter system. Packing density has been linked to the consecutive-order oil sorption efficiency. The highest diesel absorbed was attributed to the highest packing density of 0.097 g/cm^3^. This resulted in a stronger contact between the hydrophobic oil molecules and the sorbent due to the presence of a larger number of active sites on the surface of CP (acyl long chain functional groups) for the diesel to be absorbed [13]. Meanwhile, the diesel concentration filtered through the column significantly affects the efficiency of the diesel absorbed by the packed CP.

On the other hand, the factor insignificant to the response was the time of heating (A). The duration did not affect the response due to the structural modifications that occurred during heating CP at 200 °C. No interaction was observed for all of the factors affecting the diesel absorbed by CP. The F-value of 13.94 indicates that the model is significant with a coefficient of R^2^ of 0.9262. The coded factors for a second-order polynomial equation were generated based on the multiple regression analysis as follows:

Y= +48.45 − 1.83A + 15.87B + 4.46C − 2.27AB − 2.53AC + 1.85BC − 8.12A^2^ + 0.5899B^2^ − 0.6475C^2^ (2)

**Figure 9:**
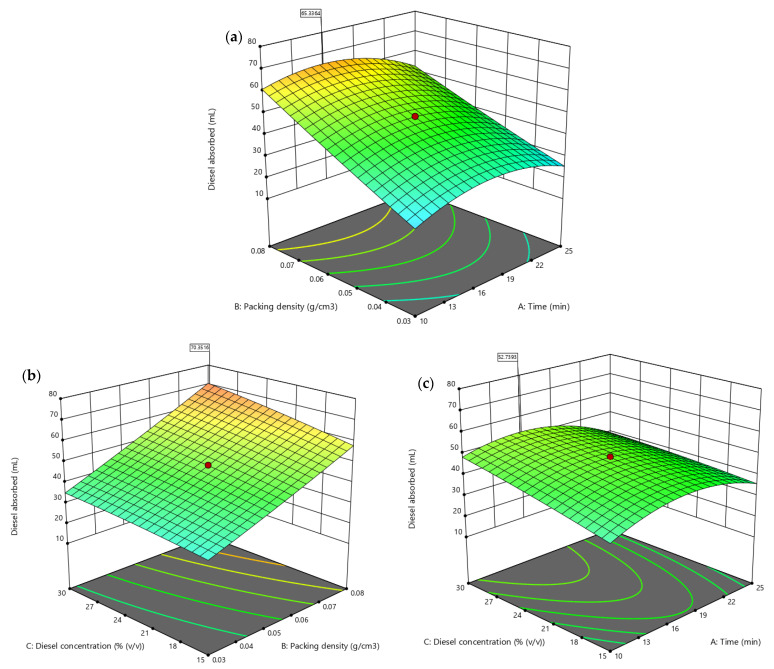
Three-dimensional response surface contour plots generated through Design Expert 13.0 (Stat Ease, Inc., Minneapolis, MN, USA.) displaying the interacting model terms; (**a**) A: time and B: packing density, (**b**) B: packing density and C: diesel concentration, and (**c**) A: time and C: diesel concentration.

A 3D contour plot was used for the CCD responses to represent the important interactions between two variables and the responses while keeping other factors constant [59,60,61]. Figure 9a illustrates the interaction between time and packing density with a fixed diesel concentration at 22.5% (*v*/*v*). The highest diesel absorbed was observed 65.33 mL by CP between the time 15 and 22 min with a maximum packing density of 0.08 g/cm^3^. Figure 9b shows the interaction between diesel concentration and packing density while maintaining constant time (17.5 min). The diesel absorbed was the highest at 70.35 mL with a maximal packing density of 0.08 g/cm^3^ and a diesel concentration of 30% (*v*/*v*). Figure 9c displays the interaction of heating time and diesel concentration with a constant packing density of 0.08 g/cm^3^. The maximum diesel absorbed (53.74 mL) was revealed at 30% (*v*/*v*) of diesel concentration between 13 and 22 min.

### 2.5. Model Validation and Performance Analysis

In a statistical optimisation of various factors, the conventional OFAT technique is less feasible, time-consuming and unable to observe any interactions between the parameters. The RSM approach ensures the optimisation of all given significant factors by providing interactions between the factors and responses. Additionally, based on the comparison between experimental and predicted values, the deduction on the variables could be accurately determined with a minimal number of experimental runs and errors [53,60,62].

The optimal values predicted by RSM are shown in Table 5. Validation was performed on the given conditions optimising the diesel sorption by CP. The experimental value on the efficiency of diesel absorbed resulted in 59.17% (71.00 ± 0.71 mL), validating the model with no significant difference (*p* = 0.4324), as determined via a two-sided Fisher’s test. Table 6 shows the comparison of the model’s performance through OFAT and RSM approaches. The diesel absorbed CP was higher in RSM than OFAT under optimal conditions. However, the efficiency of diesel absorbed was slightly higher in RSM, with a difference of 0.84%, and no significant difference was identified (*p* > 0.05). The duration of heat treatment (14.1 min) was reduced in RSM while the packing density and diesel concentration were set at the maximum. Combining these optimal conditions greatly enhanced the interactions among the significant variables in diesel sorption effectiveness by CP.

## 3. Materials and Methods

### 3.1. Materials

Agriculture waste CP (7 kg) was obtained from a local agriculture products distributer, Merbau Agrotechnology (M) Sdn Bhd, Merlimau, Melaka, Malaysia. The coco peat (CP) was separated and sieved using 5 mm × 5 mm square plastic 12-mesh wire and distributed into coco peat (CP), coco fibre (CF) and coco peat mix (1:1 ratio of fibre: peat) (CPM). Diesel fuel (PETRONAS *Dynamic Diesel*) was bought from the nearby PETRONAS fuel station UPM Serdang, Selangor, whereas the seawater was acquired at Port Klang (2.9999° N, 101.3928° E), Selangor, Malaysia (salinity = 15–19 ppt, pH = 7.5–8.1).

### 3.2. Laboratory Scale Set-Up and Sorbent Selection

Physical treatment through heat was applied to each sample (CP, CF and CPM) at 200 °C for 20 min. All heat treatments were conducted using a laboratory Taisite drying oven (forced convection oven (FCO)) with an accuracy of ±1 °C. (Taisite Lab Sciences Inc., New York, NY, USA). Both untreated and treated samples were stored in dry resealable bags with silica to prevent excessive moisture build-up ahead of use. The screening was executed to test the sorption capacity, efficiency of diesel and seawater absorbed among these samples at room temperature of 20 °C (±5 °C). A PET plastic bottle without a cap (400 mL) was used as the filtration column with the bed height at 250 mm × 50 mm and fixed to the retort stand. A cylindrical spacer (h = 6.5 cm and d = 5 cm) made from PVC mesh wire (0.15 mm × 0.15 mm) was placed in the column at the height of 20 cm. The spacer weight was ensured to not outweigh the mass of the sample. A 10% (*v*/*v*) mixture of 40 mL of diesel was mixed with 400 mL of seawater in a 1000 mL beaker. Five grams of each of CP, CF and CPM were packed into the cylindrical spacer, and the diesel–seawater mixture was poured into the filtration column. The sample was allowed to drip (gravitational pull) for 10 min, the sample wetted with diesel–seawater was weighed and the effluent volume was recorded. CP was shown to be ideal and effective in diesel sorption among the samples screened and therefore utilised for corresponding experiments. The sorption capacity (Equation (1)) according to the standard protocol, the American Society for Testing and Materials (ASTM) F726-17, and the efficiency of diesel and seawater absorbed (Equation (2)) were calculated [63,64]:

(3) $$\mathrm{Diesel}\;\mathrm{sorption}\;\mathrm{capacity}\;(\%)\;=\frac{S_{b}-S_{a}}{S_{a}}$$

where *S_b_* is the mass of a diesel-wetted sample and *S_a_* is the mass of the sample before filtration

(4) $$\mathrm{Efficiency}\;\mathrm{of}\;\mathrm{diesel}/\mathrm{seawater}\;\mathrm{absorbed}\;(\%)\;=\frac{E_{a}-E}{E_{a}}\;\times \;100$$

where *E_a_* is the initial volume of diesel/seawater and *E* is the volume of diesel/seawater removed through filtration.

### 3.3. Sorbent Characterisation

#### 3.3.1. Fourier Transform Infrared Spectroscopy (FTIR) Measurement

Infrared spectra of functional groups of the untreated and treated CP before and after filtration were measured using FTIR (ALPHA, Bruker Optik GmbH, Ettlingen, Germany) through the attenuated total reflection (ATR) mechanism. The FTIR spectra were scanned between the wavenumbers ranging from 500 to 4000 cm^−1^.

#### 3.3.2. Morphological Analysis

Morphological appearances of CP samples were observed by Variable Pressure Scanning Electron Microscopy (VPSEM) (Zeiss LEO 1455, Carl Zeiss AG, Oberkochen, Germany), and the composition of elements was identified through Energy Dispersive X-ray (EDX) microanalysis (INCA V5.2, Oxford Instruments plc, Oxfordshire, UK). CP particles were mounted on aluminium stubs (12 mm diameter) using conductive carbon tape and sputter-coated with gold using Auto Fine Sputter Coater (JEC-3000 FC, JEOL Ltd., Tokyo, Japan) for 3 min. Mounted stubs samples were accommodated on the VPSEM stage operated at a probe current of 12–13 μA and beam voltage of 5–20 kV and viewed in varying magnification ranges. In the EDX analysis, the gold element was established as the standard for beam optimisation and spectrums indicate the elements detected in the samples.

### 3.4. Statistical Optimisation

Analysis of optimised factors affecting the efficiency of diesel–seawater sorption were carried out through two stages: primary optimisation by One-Factor-At-a-Time (OFAT) and secondary screening for relevant variables through Plackett–Burman design (PBD) followed by second-order Box–Wilson’s Central Composite design (CCD) via Response Surface Methodology (RSM).

#### 3.4.1. Evaluation of Optimal Effects on the Efficiency of Diesel–Seawater Sorption Using OFAT

Various optimal factors affecting the diesel–seawater sorption capacity and efficacy were designed through OFAT by manipulating a single factor while ensuring others were constant. The effects and their experimental values were accordingly optimised to temperature (°C): 170, 180, 190, 200 and 210; time (min): 5, 10, 15, 20 and 25; packing density (g/cm^3^): 0.03, 0.04, 0.05, 0.06, 0.07 and 0.08; and diesel concentration % (*v*/*v*): from 5, 10, 15, 20, 25 and 30. The experiments were conducted in triplicates, and significance was reported through one-way analysis of variance (ANOVA) along with pairwise post hoc assessments using Tukey’s test using GraphPad Prism 8.0.2 software (GraphPad Inc., San Diego, CA, USA) to analyse the effects of each factor on the sorption capacity and efficiency of diesel and seawater absorbed.

#### 3.4.2. Plackett–Burman (PB) Design

The objective of the design is to identify important factors among a broad set of attributes and highly confounded. The PB design was adopted to select the significant factors for evaluating diesel–seawater efficiency. All four effects were selected: temperature (°C), time (min), packing density (g/cm^3^) and diesel concentration % (*v*/*v*). The design resulted in 18 individual experimental runs, including 6 centre points. To establish the settings that were nearest to optimal, each component was examined at two levels: low (−1) and high (+1) of its corresponding values (Table 7). Significant (*p* < 0.05) values were determined to profoundly impact the efficiency of diesel–seawater sorption and further analysis using CCD [65,66,67,68]. The PB factorial design at two levels was described by the following equation (Equation (3)):

(5) $$Y=\beta_{0}+\overset{k}{\underset{i=1}{\sum }}\beta_{i}{Χ}_{i}$$

where Y is the efficiency of diesel and seawater absorbed (responses), $\beta_{0}$ is the intercepted model, $\beta_{i}$ is the coefficient of linearity, ${Χ}_{i}$ is the independent variable’s coded level and k is the number of variables [67,69].

#### 3.4.3. Central Composite Design (CCD)

Based on the regression analysis, three selected factors were used: time (min), packing density (g/cm^3^) and diesel concentration % (*v*/*v*) with *p* < 0.05 to identify the effects on diesel and seawater sorption. In CCD, the numeric factor was assessed at five levels: +α and −α (axial points), +1 and −1 (factorial points), and centre point [59,69]. The alpha points described by the distance between the centre point and the axial runs in the coded scale were selected based on rotatable (*k* < 6). The output design resulted in 20 experimental runs with three significant factors (Table 8). To define the interaction between the efficiency of diesel and seawater absorption (responses), and independent variables, the quadratic CCD model was developed based on the second-order polynomial equation (Equation (4)) as follows:

(6) $$Y=\beta_{0}+\overset{k}{\underset{i=1}{\sum }}\beta_{i}{Χ}_{i}+\overset{k}{\underset{i=1}{\sum }}\beta_{ii}{Χ}_{i^{2}}+\overset{k}{\underset{1=i\text{<}j}{\sum }}\beta_{ij}{Χ}_{i}{Χ}_{j}$$

where Y is the efficiency of diesel and seawater absorbed (responses), all ${Χ}_{}$ represent independent variables at the coded level, $\beta_{0}$ is model intercept, $\beta_{i}$ is linear coefficient of ith, $\beta_{ii}$ is the quadratic coefficient, $\beta_{ij}$ is the coefficient of interaction and k is the number of factors. ANOVA determined the significance of the model and regression coefficients. This was followed by statistical analysis using Fisher’s statistical test (F-test) to analyse the significance of each independent variable. The interaction between significant factors was evaluated using response surface and 3D contour plots of predicted model responses [58,61]. All PB and CCD experimental runs were conducted in triplicates with mean and standard error measures (SEM) further analysed using Design Expert 13.0 software (Stat-Ease Inc., Minneapolis, MN, USA).

## 4. Conclusions

Coco peat as a potential biosorbent for ex situ oil-spill clean-up was assessed through a lab-scale-based filter system. Different conditions influenced the selectivity and efficiency of the diesel absorbed. The RSM approach exhibited greater efficiency of the diesel absorbed at 59.17% (71.00 mL) than OFAT (58.33%). Statistical optimisation on the efficiency of diesel absorbed by CP enclosed the significant factors (time, packing density and diesel concentration) and possible interactions affecting the responses. There is a great deal of interest in using sorbents based on natural materials due to their low cost, environmentally friendly nature, ease of deployment and high efficiency.

## Figures and Tables

**Table 1:** Secondary screening of significant parameters for diesel–seawater sorption by CP using Plackett–Burman design (±SEM, *n* = 3).

Run	A: Temperature (°C)	B: Time (min)	C: Packing Density (g/cm^3^)	D: Diesel Concentration % (*v*/*v*)	Diesel Absorbed (mL)	Seawater Absorbe (mL)
1	190	10.0	0.080	15.0	50.00 ± 0.00	21.67 ± 4.08
2	190	10.0	0.030	30.0	31.67 ± 5.40	3.33 ± 2.04
3	190	25.0	0.030	30.0	35.00 ± 0.00	5.00 ± 0.00
4	210	25.0	0.030	15.0	3.00 ± 0.00	25.00 ± 0.00
5	210	10.0	0.080	30.0	65.00 ± 1.41	10.00 ± 0.00
6	200	17.5	0.055	22.5	53.67 ± 2.48	8.33 ± 2.04
7	200	17.5	0.055	22.5	53.67 ± 2.48	8.33 ± 2.04
8	200	17.5	0.055	22.5	53.67 ± 2.48	8.33 ± 2.04
9	190	25.0	0.080	15.0	55.00 ± 0.00	13.33 ± 2.04
10	190	25.0	0.080	30.0	54.33 ± 0.81	8.33 ± 2.04
11	190	10.0	0.030	15.0	22.00 ± 1.22	11.00 ± 1.22
12	200	17.5	0.055	22.5	53.67 ± 2.48	8.33 ± 2.04
13	210	25.0	0.080	15.0	0.00	65.00 ± 0.00
14	210	25.0	0.030	30.0	10.00 ± 0.00	25.00 ± 0.00
15	210	10.0	0.030	15.0	30.33 ± 0.41	6.67 ± 2.04
16	200	17.5	0.055	22.5	53.67 ± 2.48	8.33 ± 2.04
17	210	10.0	0.080	30.0	70.33 ± 4.51	5.00 ± 3.19
18	200	17.5	0.055	22.5	53.67 ± 2.48	8.33 ± 2.04

**Table 2:** ANOVA analysis to determine the significant factors affecting the diesel sorption by CP.

Source	Sum of Squares	df	Mean Square	F-Value	*p*-Value	
Model	4587.30	4	1146.82	8.43	0.0018 *	significant
A-Temperature	400.59	1	400.59	2.95	0.1118	
B-Time	1045.33	1	1045.33	7.69	0.0169 *	
C-Packing density	2205.04	1	2205.04	16.21	0.0017 *	
D-Diesel concentration	936.33	1	936.33	6.88	0.0222 *	
Curvature	1312.05	1	1312.05	9.65	0.0091	
Residual	1632.11	12	136.01			
Lack of Fit	1617.89	6	269.65	113.76	<0.0001 ***	significant
Pure Error	14.22	6	2.37			
Cor Total	7531.46	17				
Std. Dev.	11.66		R^2^	0.7376		
Mean	41.59		Adjusted R^2^	0.6501		
C.V. %	28.04		Predicted R^2^	0.2288		
			Adeq Precision	9.4226		

* *p* < 0.05, ** *p* < 0.01 and *** *p* < 0.0001.

**Table 3:** Optimisation of factors affecting diesel–seawater sorption by CP using central composite design (CCD) (±SEM, *n* = 3).

Run	A: Time (min)	B: Packing Density (g/cm^3^)	C: Diesel Concentration % (*v*/*v*)	Diesel Absorbed (mL)		Seawater Absorbed (mL)	
				Experimental Value	Predicted Value	Experimental Value	Predicted Value
1	10	0.08	15	48.33 ± 2.04	51.40	21.67 ± 2.04	19.42
2	10	0.03	15	21.00 ± 1.22	18.83	15.47 ± 0.57	15.85
3	25	0.08	15	54.33 ± 0.41	48.27	17.00 ± 2.45	20.54
4	17.5	0.055	22.5	48.67 ± 1.08	48.45	5.00 ± 0.00	5.09
5	4.88655	0.055	22.5	32.40 ± 1.59	28.57	20.67 ± 2.86	23.54
6	17.5	0.055	22.5	48.67 ± 1.08	48.45	5.00 ± 0.00	5.09
7	17.5	0.055	22.5	48.67 ± 1.08	48.45	5.00 ± 0.00	5.09
8	17.5	0.0129552	22.5	16.67 ± 0.82	23.43	2.33 ± 1.78	0.0950
9	17.5	0.055	22.5	48.67 ± 1.08	48.45	5.00 ± 0.00	5.09
10	17.5	0.055	35.1134	50.50 ± 3.08	54.11	6.67 ± 2.04	4.71
11	17.5	0.055	22.5	48.67 ± 1.08	48.45	5.00 ± 0.00	5.09
12	30.1134	0.055	22.5	11.00 ± 0.00	22.40	30.00 ± 0.00	24.01
13	25	0.03	30	33.33 ± 3.49	24.91	3.67 ± 1.63	8.11
14	10	0.03	30	28.40 ± 1.02	29.11	10.00 ± 0.00	8.67
15	17.5	0.0970448	22.5	76.00 ± 1.22	76.81	14.67 ± 3.27	13.79
16	17.5	0.055	22.5	48.67 ± 1.08	48.45	5.00 ± 0.00	5.09
17	17.5	0.055	9.88655	35.17 ± 0.82	39.12	11.67 ± 4.08	10.51
18	25	0.08	30	59.00 ± 0.71	55.81	19.00 ± 0.71	20.82
19	10	0.08	30	68.60 ± 2.87	69.08	8.33 ± 2.04	8.48
20	25	0.03	15	30.60 ± 1.77	24.76	2.00 ± 0.00	4.06

**Table 4:** ANOVA output for CCD model identifying factors and interactions significantly affecting the diesel sorption.

Source	Sum of Squares	df	Mean Square	F-Value	*p*-Value	
Model	4854.49	9	539.39	13.94	0.0001 ***	significant
A	45.97	1	45.97	1.19	0.3012	
B	3439.11	1	3439.11	88.90	<0.0001 ***	
C	271.16	1	271.16	7.01	0.0244 *	
AB	41.10	1	41.10	1.06	0.3269	
AC	51.34	1	51.34	1.33	0.2761	
BC	27.38	1	27.38	0.7078	0.4199	
A^2^	950.03	1	950.03	24.56	0.0006 **	
B^2^	5.02	1	5.02	0.1296	0.7263	
C^2^	6.04	1	6.04	0.1562	0.7010	
Residual	386.85	10	38.69			
Lack of Fit	386.85	5	77.37			
Pure Error	0.0000	5	0.0000			
Cor Total	5241.34	19				
Std. Dev.	6.22		R^2^	0.9262		
Mean	42.87		Adjusted R^2^	0.8598		
C.V. %	14.51		Predicted R^2^	0.4362		
			Adeq Precision	13.1818		

A: time, B: packing density, C: diesel concentration, * *p* < 0.05, ** *p* < 0.01 and *** *p* < 0.0001.

**Table 5:** RSM model validation using the optimal predicted values.

Parameters	Optimised Value	Efficiency of Diesel Absorbed	
		Predicted Value	Experimental Value
Time (min)	14.1	71.91 mL (59.93%)	71.00 ± 0.71 mL (59.17%)
Packing density (g/cm^3^)	0.08		
Diesel concentration % (*v*/*v*)	30		

**Table 6:** Model performance.

Parameters	Optimised Values	
	OFAT	RSM
Temperature (°C)	200	200
Time (min)	20.0	14.1
Packing density (g/cm^3^)	0.06	0.08
Diesel concentration % (*v*/*v*)	25	30
Diesel absorbed (mL)	58.33 ± 2.04	71.00 ± 0.71
Efficiency of diesel absorbed (%)	58.33 ± 2.04%	59.17 ± 0.71%

**Table 7:** PB design for experimental values and coded levels of factors analysed for the efficiency of diesel and seawater absorbed.

Factors	Units	Experimental Value	
		Low (−1)	High (+1)
A: Temperature	°C	190	210
B: Time	min	10	25
C: Packing density	g/cm^3^	0.03	0.08
D: Diesel concentration	% (*v*/*v*)	15	30

**Table 8:** CCD design for experimental values and the five coded levels of three significant factors analysed for the efficiency of diesel and seawater absorbed.

Factors	Units	Experimental Values				
		Alpha (−2)	Low (−1)	0	High (+1)	Alpha (+2)
A: Time	min	4.88655	10	17.5	25	30.1134
B: Packing density	g/cm^3^	0.0129552	0.03	0.055	0.08	0.0970448
C: Diesel concentration	% (*v*/*v*)	9.88655	15	22.5	30	35.1134

## Data Availability

Not applicable.
